# Beyond the Cuckoo’s Nest: Patient and Public Attitudes about Psychiatric Electroceutical Interventions

**DOI:** 10.1007/s11126-021-09916-9

**Published:** 2021-04-17

**Authors:** Laura Y. Cabrera, Maryssa M. C. Gilbert, Aaron M. McCright, Eric D. Achtyes, Robyn Bluhm

**Affiliations:** 1 Center for Neural Engineering, Department of Engineering Science and Mechanics, Penn State University, University Park, W-319 Millennium Science Complex, State College, PA 16802 USA; 2 Rock Ethics Institute, Penn State University, University Park, State College, PA USA; 3 College of Human Medicine, Michigan State University, East Lansing, MI USA; 4 Department of Sociology, College of Social Science, Michigan State University, East Lansing, MI USA; 5 Division of Psychiatry & Behavioral Medicine, College of Human Medicine, Michigan State University, Grand Rapids, MI USA; 6 Pine Rest Christian Mental Health Services, Grand Rapids, MI USA; 7 Department of Philosophy, College of Arts and Letters, Michigan State University, East Lansing, MI USA; 8 Lyman Briggs College, Michigan State University, East Lansing, MI USA

**Keywords:** Depression, Electroceuticals, General public, Patients, Attitudes

## Abstract

Recent research emphasizes the role of psychiatric electroceutical interventions (PEIs), bioelectronic treatments that employ electrical stimulation to affect and modify brain function, to effectively treat psychiatric disorders. We sought to examine attitudes about three PEIs—electroconvulsive therapy, transcranial magnetic stimulation, and deep brain stimulation—among patients with depression and members of the general public. As part of a larger study to assess different stakeholders’ attitudes about PEIs, we conducted semi-structured key informant interviews with 16 individuals living with depression and 16 non-depressive members of the general public. We used a purposive sampling approach to recruit potential participants based on eligibility criteria. We performed qualitative content analysis of interview transcripts. Participants from both groups expressed an overall cautionary attitude towards PEIs, yet there were mixed attitudes in both groups. Patients commonly described electroconvulsive therapy as scary, traumatic, or intense, while members of the general public often referenced the treatment’s negative portrayal in *One Flew over the Cuckoo’s Nest*. Patients and the general public saw transcranial magnetic stimulation as a potentially viable option, but in most cases only if medication was not effective. Deep brain stimulation attitudes were predominantly negative among patients and cautionary among public. The overall cautionary attitudes towards PEIs, together with the technological features and social aspects underlying those attitudes, highlight the need for unbiased education to fill the gaps in knowledge and inform perceptions of those who may benefit from these treatments.

**Supplementary Information:**

The online version contains supplementary material available at 10.1007/s11126-021-09916-9.

## Introduction

Major depressive disorder (MDD) is a serious mental health problem in the United States (US). An estimated 14.4% of adolescents and 7.2% of adults in the US experienced a major depressive episode in 2018, and this prevalence has been increasing over the past few years for both groups [1]. Antidepressants are generally recommended as first-line treatment of mild to moderate MDD [2], although other guidelines recommend psychotherapy and exercise for mild or subclinical depression [3]. However, only 36.8% of MDD patients achieve remission with the first antidepressant medication [4], and only 70% of MDD patients achieve remission after 4 treatments [5]. This leaves about 30% of MDD patients in the US in need of an alternative regimen for managing their depression. If patients do not respond to a second medication of a different pharmacological class, and both treatments have been used for a sufficient length of time at an adequate dose, their depression is considered to be treatment resistant. Treatment-resistant depression (TRD) is associated with reduced quality of life [6], as well as increased morbidity [7] and economic burden [8, 9].

At this time, electroconvulsive therapy (ECT) has the highest rates of response and remission for TRD of any form of antidepressant treatment [2]; however, some patients are not responsive to ECT [10]. Because of this, other psychiatric electroceutical interventions (PEIs) for TRD have been approved by the U.S. Food and Drug Administration (FDA) or are under investigation. Transcranial magnetic stimulation (TMS) is one FDA-approved therapy for MDD patients who have not responded to a single adequate antidepressant trial [11, 12]. A recent expert consensus statement declared that multiple randomized controlled trials support the safety and efficacy of repetitive TMS (rTMS) for treating MDD [13]. Scientists are investigating the effects of another PEI—deep brain stimulation (DBS), which is not yet FDA-approved for MDD—on several targets in the brain. While several open-label DBS trials found that the intervention produces 30–50% remission rates [14–19], the few randomized controlled trials have shown conflicting results [20], with two multi-site randomized, sham-controlled trials finding no statistically significant benefit [21, 22]. A likely future development in this intervention is closed-loop DBS (which we refer to here as an Adaptive Brain Implant or ABI), in which the stimulation can be adjusted on-demand or adapted based on fluctuating symptoms [23, 24].

Historically, psychiatric treatments have been met with uneven public reception. At times, the public rapidly endorsed certain psychiatric treatments for desperately ill patients [25]. Yet, in other cases, as with ECT [26, 27], negative attitudes persist, despite sound scientific evidence regarding the intervention’s safety and efficacy [28, 29]. Inaccurate portrayals of mental illness and its treatment in the media in general may provoke anti-psychiatry biases [30] or spread uncritical, premature acceptance and uptake of unproven treatments [31, 32]. Indeed, misinformation about, misperceptions of, and/or stigma toward PEIs in particular may undermine their acceptance and reduce referrals for their application. As such, there is a need to better understand key stakeholders’ attitudes and beliefs about using PEIs for treating MDD for the following reasons: first, patients’ and the public’s ideas about PEIs and MDD likely influence their own and/or others’ help-seeking behavior [33]; and second, accurately understanding these stakeholders’ views likely will help professionals more effectively communicate treatment recommendations.

To date, several studies have examined patient and public views about PEIs, with most focusing on ECT. Several studies found generally positive attitudes toward ECT among patient samples in New York [34], France [35], and Turkey [36]. Such favorable views persist even in the immediate post-ECT period [37]. The few studies of public beliefs and attitudes toward ECT—whether in Germany [38], Switzerland [26], or the United Kingdom [39]—find rather low levels of knowledge about ECT [38, 39] and generally negative views toward it [26, 38, 39]. McFarquhar and Thompson [39] note that approximately 70% of their sample reported having seen ECT portrayed in movies, with *One Flew Over the Cuckoo’s Nest* being the one most frequently mentioned.

To our knowledge, no published research has examined public views toward TMS, DBS, or ABIs. Yet, a few studies have analyzed patient attitudes toward TMS in Australia [40], Israel [41], and the US [42]. They found that patients generally had positive attitudes toward TMS [40, 41], especially with increased knowledge about the PEI [42]. Other studies have investigated how patients with MDD in the US [43] and patients with obsessive compulsive disorder (OCD) in the Netherlands [44] perceived DBS. While patients in the former study reported positive attitudes toward DBS, those in the latter study—who actually had a DBS implant—disclosed that they accepted DBS as a last resort only after other treatments were ineffective. Finally, one study examined how patients with DBS implants perceived closed-loop or next-generation DBS devices (i.e., ABIs) [45]. These subjects expressed optimism that closed-loop technology could improve upon certain limitations of open-loop devices, particularly the maintenance burden of traditional open-loop treatment systems, such as battery replacements.

To contribute to this emerging literature, as part of a larger project examining different stakeholder attitudes to PEIs use for treatment resistant depression (TRD), we interviewed a sample of patients and members of the public about their attitudes, perceptions, and concerns about three PEIs: ECT, TMS, and DBS.

## Methods

We conducted key informant interviews with 16 adult MDD patients and 16 adults in the general public with no diagnosed psychiatric disorder. Key informant interviews are ideal for eliciting in-depth, meaningful information from subjects [46]. In our analysis below, we focused on these groups’ views on ECT, TMS, and DBS.

### Recruitment

We recruited potential participants in mid-Michigan via flyers in mental health clinics, local establishments (e.g., coffee shops and libraries), and social media posts. In addition to the eligibility criteria above, we used a purposive sampling approach with quotas to recruit into our two groups. We used quotas for gender and race in each group to ensure demographic diversity. Table 1 provides key demographic and social characteristics of our two groups. In our patient group, we also employed a quota for PEI experience, so at least five patients had at least moderate experience with a PEI. In our public group, we employed a quota for caregiving experience, so at least five adults in the public group had moderate or greater experience caregiving for a family member or close friend with a psychiatric disorder. The top section of Table 2 defines these levels of PEI and caregiving experience. (See Tables SDC1 and SDC2 in the Supplemental Digital Content for key characteristics of the sampled patients and members of the public, respectively.)

**Table 1:** Description of the Study Samples

	Patients (n = 16)	Public (n = 16)
Gender		
Male	6 (37.5%)	7 (43.8%)
Female	10 (62.5%)	9 (56.3%)
Age		
Median	48.5	40
Range	18–65	20–81
Race/Ethnicity		
Latino White	1 (6.3%)	5 (31.4%)
non-Latino White	12 (75.0%)	9 56.3%)
African American	2 (12.5%)	0 (0.0%)
Asian American	1 (6.3%)	2 (12.5%)
Education		
High school	3 (18.8%)	1 (6.3%)
Some college	2 (12.5%)	3 (18.8%)
Bachelor’s degree	5 (31.4%)	8 (50.0%)
Master’s degree	5 (31.4%)	3 (18.8%)
Doctoral degree	1 (6.3%)	1 (6.3%)
Marital Status		
Single	7 (43.8%)	5 (31.4%)
Married	4 (25.0%)	9 (56.3%)
Divorced	4 (25.0%)	1 (6.3%)
Widowed	1 (6.3%)	1 (6.3%)

**Table 2:** Participants’ Experiences and Attitudes

Experience with PEI/Caregiving
*Substantial*: Patient has personally used one or more PEIs for several years Member of public has provided physical and emotional support to close family member or friend, including driving them to appointments, helping them with activities of daily living, and helping them take their medication
*Moderate*: Patient has started to use PEI recently Member of public has provided physical and emotional support to close family member or friend but not in a daily basis
*Minimal*: Patient has only heard of PEI Member of public has only provided emotional support to a family member or friend (e.g., listening to them if they needed to talk, grabbing dinner, or discussing their mood and depression symptoms)
Attitude toward PEI
*Positive*: Participant expressed largely optimistic or hopeful views about PEI Participant voiced mostly positive comments about PEI
*Cautionary*: Participant did not reject the PEI, but voiced concerns about its risks Participant viewed PEI only as last resort or after learning about all other options Participant expressed skepticism about PEI’s effectiveness or felt it needed further development
*Negative*: Participant expressed clear, pronounced criticism of PEI Participant voiced mostly negative comments about PEI

### Compliance with Ethical Standards

Our study received a human subjects exemption from the Institutional Review Board at Michigan State University (STUDY00001247). This exemption allowed us to secure oral, rather than written, consent from participants. When scheduling interviews, we sent participants a consent letter that contained key details about the research study. (See Supplemental Digital Content 3 and 4 for the consent letters of patient and public interviews, respectively.) We also sent them a brief fact sheet that summarized the PEIs that our overall project focuses on and included details about their FDA-approval status for the treatment of MDD. Prior to each interview, we invited participants to ask any questions they had about our study before securing their oral consent to participate.

### Procedures and Survey Instrument

Consistent with the exploratory nature of qualitative work, we employed semi-structured interview guides to cover a core set of topics while probing for in-depth understanding and exploring unanticipated and/or emerging issues (Miles et al., 2013). We created our interview guides using insights from prior relevant studies and feedback from our project’s Scientific Advisory Board. (See Supplemental Digital Content 5 and 6 for the semi-structured guides for the patient and public interviews, respectively.) The interview length ranged from 30 to more than 60 min, with an average of 48 min. Participants were compensated with a $50 gift card for their time.

A team member conducted each semi-structured interview in person, via Zoom, or by telephone. After some general questions about participants’ experience with PEIs, participants answered detailed questions about either one or two PEIs (depending on time limits). Interviewers selected the specific PEIs for each participant based on their experience and interest, while also keeping a balance across the PEIs in the study. Since ABIs are relatively novel and still under development, we asked only a few questions about them and have excluded them from this analysis. While these interviews covered a range of PEI topics, we focus this paper on patients’ and the public’s attitudes towards PEIs.

### Data Management and Analysis

We recorded, anonymized, and then transcribed the content of each interview. We performed our content analysis using the web-based software Dedoose, which allows for rigorous qualitative analysis within a mixed-methods framework. A priori codes were based on the topics covered in interview questions and from the neuroethics literature on these topics. Using qualitative content analysis methods and a deliberative approach [47, 48] we analyzed the first few transcribed interviews to create a draft codebook and identify emergent issues that warranted further exploration in subsequent interviews. We then analyzed a few additional transcripts to apply any necessary adjustments to the codebook. The team then convened to reach consensus on the coding framework. Two team members coded the remaining interviews with the finalized codebook, and another two served as secondary coders. Team meetings provided further opportunities to revise the codebook as necessary (e.g., refining, merging, and/or distinguishing themes and sub-themes) and to help the team reach consensus on any coding discrepancies.

In addition to identifying emergent themes and sub-themes, we also coded participant’s attitude toward each PEI discussed in detail as positive, cautionary, and negative—as described in the bottom section of Table 2. While participants may have expressed a mix of these attitudes, we identified the predominant attitude for a given PEI throughout each interview. Table 3 presents the percentages of positive, cautionary, and negative attitudes about each PEI across the 32 interviews. (See Table SDC3 in the Supplemental Digital Content for each participant’s predominant attitude about each PEI, as applicable.) When presenting our results, we include interview excerpts to illustrate key themes and sub-themes. With each excerpt, we include the anonymized participant number. For readability, we have removed any non-content utterances.

**Table 3:** Predominant attitude toward PEIs

	ECT		TMS		DBS		Total	
Attitude	Patients	Public	Patients	Public	Patients	Public	Patients	Public
Negative	35.7%	33.3%	11.1%	0.0%	46.2%	7.7%	33.3%	13.8%
Cautionary	57.1%	33.3%	66.7%	57.1%	30.8%	61.5%	50.0%	51.7%
Positive	7.1%	33.3%	22.2%	42.9%	23.1%	30.8%	16.7%	34.5%
N	14	9	9	7	13	13	36	29

## Results

### Cautionary Attitudes about PEIs

Across all PEIs combined, 50.0% of patients’ responses and 51.7% of the public’s responses were cautionary. Four themes were associated with such a cautionary attitude. First, some people expressed concern about how directly PEIs affect the brain compared to how medications or psychotherapy work.

“[A PEI in general] is a little different because it’s affecting the brain. (Laughs) Which is the whole mass, circuitry. It’s the whole, you know, computer of the body.” (Public 5)

“[DBS] just seems to be more direct, so you’re not dealing with all of [the medication] side effects, stuff going in your blood stream. ... but yeah, the downside would be you’re messing with a brain; that’s a pretty big deal.” (Public 14)

A related theme was participant’s concern about the risks with, or the potential side effects of, these interventions. Patients especially reported concern about the potential for memory loss with ECT.

“I worry every time. Am I zapping more of my brain cells? And, you know, what won’t I be able to get back?” (Patient 12)

“I think that a potential risk might be that it changes your brain for good. And for the worst for good. You know, like the memory loss. [T]hey say “short-term memory loss” and it’s not permanent. Well, what if it is permanent?” (Patient 13)

With DBS, participants noted risks related to undergoing brain surgery as well as having an implant on their brain.

“Brain surgery is always a big risk. Risk that you are undergoing the surgery and then it doesn’t actually help, which is a big, big emotional and financial burden.” (Public 7)

“I would be afraid of maybe the materials because if they are inside my head, not only on the surface, they can have a secondary effect.” (Public 9)

Even with TMS, where the potential side effects are not as serious, both patients and members of the public mentioned them as a reason for being cautious.

“[T]he rare but serious side effects…. somewhat turn me off because it just seems like if it could [happen], it’ll happen to me.” (Patient 15)

“[T]hey’re stimulating the brain, so there is always a chance that something could be affected.” (Public 10)

A third theme associated with a cautionary attitude was concern about invasiveness.

“I’d say [ECT is] very invasive because it’s getting down to the physical brain in itself and sending signals directly to the brain where everything is happening.” (Patient 11)

“[T]here is something different about putting something inside of my brain as opposed to putting something out here. [points to scalp]… [T]he degree of invasion is a major concern for me as a patient.” (Patient 4, discussing DBS)

However, for a few members of the public, a brain implant’s similarity to a cardiac pacemaker made its invasiveness less unsettling.

“Even though it’s invasive, for some reason [DBS] felt like it was okay because it appeared to be like a pacemaker to me.” (Public 13)

The last theme associated with a cautionary attitude was participants’ views of PEI use only as a last resort if no other alternative was available and, for some participants only after they researched the intervention more.

“If I were suicidal, I guess I would [try TMS]. I guess if I had just got to the end of my rope and felt like nothing was working, which I am close to right now because I have been taking medications for a long time and not really getting any relief.” (Patient 7)

“I think I always had sort of a negative view of ECT, but after reading about the different types of therapies that are being developed, I think I have more of a positive outlook or, ‘Oh, that’s really interesting. I want to see where that’s going,’ especially with [TMS and DBS].” (Public 2)

“I don’t know if the risk is worth it unless like—I mean I guess if nothing else is working and you’re still on the same route with the other two, [DBS] may be the next step up.” (Public 3)

In the case of TMS, some participants’ uncertainty about its effectiveness was at the core of their cautionary attitude.

“TMS was not very effective overall. I mean it helped maybe a tiny bit with my mood, but, in general, it wasn’t all that effective.” (Patient 6)

“I’m really intrigued by like what TMS could do because I don't know anything about it. …[A]m I skeptical of it? Yeah, a little bit.” (Public 4)

### Negative Attitudes about PEIs

Across all PEIs combined, 33.3% of patients’ responses but only 13.8% of the public’s responses were negative. Three themes were associated with such a negative attitude. Most participants espousing a negative attitude expressed a decidedly fearful emotional response, often describing the intervention as scary, traumatic, or intense.

“Well, [ECT] sounds like it would be pretty traumatic almost.” (Patient 2)

“I would have had a hard time saying yes to those types of therapies [DBS]… [It] sounds so invasive and scary. I hate to say this, but when you’re in that place, it’s like, ‘Just forget about it. It’s not even worth it’.” (Patient 1)

“It still feels like potentially a harmful thing…. and primitive. Even though I do believe [ECT] is safer than it used to be.” (Public 6)

A second theme associated with a negative attitude was reference to negative media or public portrayals over the years. This theme was especially found in discussions of ECT.

“I think I had more of almost a negative view from the stuff that I have seen in the media about electrical therapies [….] So the one I was thinking about was *The One Who Flew Over the Cuckoo’s Nest.*” (Public 2)

“I have a lot of concerns about [ECT]. Even though I know that’s just a popular image I’m responding to, it just reminds me of the idea of torture.” (Patient 7)

“It seems very intense and the way they portray [ECT], they fry your brain and stuff like that, which I am sure is not true 100% so. Yeah, so I’ve seen it kind of in a negative way.” (Patient 8)

**“**[B]ecause of my age, I think that what I immediately thought of was the Kennedys and the daughter that they had done, the lobotomy too, and then she ended up being a completely different person than she was.” (Public 13, referring to PEIs in general)

Finally, a theme specific to DBS associated with a negative attitude was worries about invasiveness and aversion to having something implanted in their brain.

“[DBS] sounds very invasive where they implant coils or things actually in the brain and then, you put some kind of pads on your chest or implant those, and it just seemed like it does the actual stimulus to the brain and that sounds very scary.” (Patient 1)

“I just don’t like the idea of something like that being put in my brain. It’s just sci-fi kind of scary.” (Patient 7)

### Positive Attitudes about PEIs

Across all PEIs combined, 16.7% of patients’ responses and 34.5% of the public’s responses were positive. Three themes were associated with such a positive attitude. First, some participants emphasized what they believed was the PEI’s capability to address symptoms faster and more effectively than psychotherapy or medications.

“It seems like it’s a lot faster in the sense that it would be useful for people who were at high-risk of like suicide or something like that. So ECT could be a quick response if you need it to be.” (Public 1)

“I don’t know how many people ECT works for, but it seems to continue to be an effective treatment when other things don’t work.” (Patient 12)

“I believe that [DBS] would be more effective than seeing a counselor and taking medication.” (Public 13)

Another theme was related to participants reporting that they liked how TMS was not as physically invasive or intense as were ECT or DBS.

“[TMS] didn’t sound nearly as invasive. It sounded fairly safe and without side effects.” (Patient 1)

“I wouldn’t think it’s that invasive because they’re doing it like outside your brain even though they are stimulating your brain.” (Public 10)

A final theme associated with a positive attitude, and specific to DBS, was its ability to target a specific brain region, its continuous effect, and its potential to reduce daily medications.

“[T]here might be a little bit more optimism in some of these that you mentioned [DBS and ABI] because of the directness, and it’s not a pill, it’s not something daily, it’s working targeting the brain literally [laughs].” (Patient 3)

“[B]ecause [DBS is] under the skin, it can continually be effective instead of being like a one-time thing…it just seems like it can be more beneficial over a long period of time.” (Public 1)

## Discussion

Examining attitudes about specific PEIs reveals the likely social and technological factors influencing these attitudes. For example, the routine use of electricity in everyday medical practice (e.g., electrocardiography, electroencephalography, pacemakers, treating movement disorders with DBS), suggests the possibility for some level of acceptance with its use in PEIs. Yet, participants in this study did not perceive the use of electricity for treating psychiatric conditions in the brain in a clearly positive light. The perceived uniqueness of the brain might explain patient and public participants’ predominant cautionary attitude toward using PEIs for depression. The brain is considered the seat of the self and the organ orchestrating all of our bodily functions. As such, interventions that directly affect the brain may be seen with more caution than interventions on other organs [49].

Participants who expressed a cautionary attitude recognized the benefits and the drawbacks of these interventions. Not surprisingly, most of these participants accepted PEIs only as a last resort after other treatments failed, even while acknowledging the merits of these interventions. This finding is consistent with similar attitudes about psychiatric neurosurgery interventions, including DBS, found in other studies [50].

Concern about risks and side effects was frequent among participants expressing cautionary attitudes. Participants reporting such attitudes were most worried about memory loss with ECT and the need for neurosurgery with DBS. For TMS, which is generally seen as having a mild risk profile and being not very invasive, these participants most frequently questioned its effectiveness. This may be because few patient and public participants in the study were not aware of or familiar with TMS, which was surprising given its FDA approval for treating MDD, and the subsequent expansion of TMS clinics [11].

Participants who expressed a positive attitude toward PEIs often noted that these interventions seemed superior to psychotherapy or medications. They emphasized that PEIs can directly intervene on the brain quickly and effectively, reducing the need for waiting for months or years to see results (as with psychotherapy) or having to take pills daily (as with medications). In a few cases, familiarity with implantable technologies using electricity in the body, such as pacemakers, helped some participants view DBS as a better option than ECT or TMS. Indeed, when specifically considering ECT, only 7.1% of patient participants and 33.3% of public participants expressed a predominantly positive attitude toward this well-established PEI.

Negative attitudes also had a varied origin. The degree of physical invasiveness was a major theme, whether participants worried about having an electrode implanted in their brain with DBS or undergoing general anesthesia with ECT. This perceived invasiveness seemed to be related to the fear and total rejection of PEIs among some participants.

In addition to technological features, negative attitudes toward ECT in particular seemed to be a deeply rooted response to inaccurate and sensationalistic media portrayals of the therapy. Similar to other studies [26, 38, 39], several participants specifically mentioned the movie *One Flew over the Cuckoo’s Nest*, in which hospital staff deliver ECT to the protagonist R. P. McMurphy as a means to control him. These participants viewed ECT as scary or traumatic; at least some patients even thought that the treatment was no longer available.

Negative attitudes toward ECT also seemed to reflect participants’ perceived history of the therapy. ECT has been controversial since its inception [51, 52], with much public fear due not only from prejudice but also from societal memories of ECT and other biological psychiatric interventions used for social control [25, 53]. Because of this history, contemporary proponents of ECT have tended to emphasize that the technology has been further developed and improved and that the abuses were in the distant past [54].

These results highlight the need for further research into what shapes attitudes toward PEIs among both patients and the general public—as well as among other stakeholders like caregivers and psychiatrists themselves. Such future research may build upon insights from this and other studies to more systematically investigate different stakeholders’ views about PEIs with a larger, nationally representative sample.

These results also suggest an opportunity gap for psychiatrists and other mental health practitioners to continue accurately educating members of the general public as well as potential and existing patients about the merits and risks of PEIs. At the very least, psychiatrists’ efforts may be beneficial if they effectively counter or debunk the ongoing negative influence of inaccurate or biased portrayals of ECT in the media and within our culture more broadly. Indeed, existing evidence suggests that patients receiving some education about ECT more positively perceive the therapy and are more willing to receive it [55].

## Limitations

Participants in this study self-selected to be interviewed, all lived in Mid-Michigan, and most had relatively high levels of education. As such, we caution against inferring generalizability of our results to depressed patients or the general public more broadly. At the same time, qualitative interviews are valuable for exploring participants’ thought processes and achieving greater understanding of their views. We were not able to ask each participant about all of the PEIs because of time constraints. Another factor that limited the comparability across participants was our use of semi-structured interviews. While we asked all participants the same set of core questions, their rich responses often were elicited from follow-up and probing questions.

## Supplementary Information

Below is the link to the electronic supplementary material.

Supplementary file1 (DOCX 68 KB)

## Data Availability

Access to interview data is available upon request.
