# Supplementary material for: Beyond the Cuckoo’s Nest: Patient and Public Attitudes about Psychiatric Electroceutical Interventions
Source: Psychiatr Q. 2021 Apr 17;92(4):1425–38. doi: 10.1007/s11126-021-09916-9 (PMC8531080; doi:10.1007/s11126-021-09916-9)
Supplement: Supplementary file 1 — Supplementary file1 (DOCX 68 KB) [file 11126_2021_9916_MOESM1_ESM.docx]

**Supplemental Digital Content**

**Page**

Supplemental Digital Content 1—Table SDC1 2

Supplemental Digital Content 2—Table SDC2 3

Supplemental Digital Content 3—Consent Letter for Patient Interviews 4

Supplemental Digital Content 4—Consent Letter for Public Interviews 5

Supplemental Digital Content 5—Semi-Structured Interview Guide for Patients 6

Supplemental Digital Content 6—Semi-Structured Interview Guide for Public 10

Supplemental Digital Content 7—Table SDC3 14

**Table SDC1**. Patient Experience with PEIs

**Identifier ECT Experience TMS Experience DBS Experience**

Patient1 none moderate none

Patient2 none substantial none

Patient3 none none none

Patient4 none none none

Patient5 none none none

Patient6 substantial none none

Patient7 none none none

Patient8 none none none

Patient9 none none none

Patient10 none none none

Patient11 none none none

Patient12 substantial none minimal

Patient13 none none none

Patient14 moderate moderate none

Patient15 none none none

Patient16 substantial none substantial

**Table SDC2**. Public Experience with Caregiving and Knowledge of PEIs

**Caregiving ECT TMS DBS**

**Identifier Experience Knowledge Knowledge Knowledge**

Public1 minimal minimal none none

Public2 minimal minimal none none

Public3 none none none none

Public4 minimal minimal none minimal

Public5 none minimal none none

Public6 minimal substantial moderate none

Public7 moderate substantial moderate none

Public8 none minimal minimal minimal

Public9 minimal none none none

Public10 minimal none none none

Public11 minimal minimal none none

Public12 substantial none none none

Public13 substantial minimal none none

Public14 substantial minimal minimal none

Public15 minimal none none none

Public16 substantial minimal none none

**Consent Letter for Patient Interviews**

You are being asked to participate in a research study. The purpose of the study is to identify concerns, beliefs, and attitudes about different types of treatments for clinical depression. This initial stage of our study will help us better understand what people with depression think about therapies that treat depression by using electrical stimulation to affect and modify brain function. In addition to interviews with people with depression, we are also interviewing psychiatrists as well as members of the public without depression.

In our interviews of people with depression, we are deeply interested in your views about therapies that use electrical stimulation to treat depression, including how you perceive their benefits, risks, ethical considerations around their use and how you think these therapies may be developed further.

We want to achieve as much depth of understanding as possible in our interviews. Your honest and thoughtful answers today will also help us design a standardized survey about depression treatments that use electrical stimulation, which we will be administering to a national sample of people.

For the interview, you will be asked to answer some questions. It should take about 60 minutes. Your participation is voluntary. If you not do want to answer any of the questions, please let me know. You can also ask me to stop at any time. You must be 18 or older to participate. Is it okay if I record our interview?

This research is covered by a Certificate of Confidentiality from the National Institutes of Health. The researchers with this Certificate may not disclose or use information, documents, or biospecimens that may identify you in any federal, state, or local civil, criminal, administrative, legislative, or other action, suit, or proceeding, or be used as evidence, for example, if there is a court subpoena, unless you have consented for this use. Information, documents, or biospecimens protected by this Certificate cannot be disclosed to anyone else who is not connected with the research except, if there is a federal, state, or local law that requires disclosure (such as to report child abuse or communicable diseases but not for federal, state, or local civil, criminal, administrative, legislative, or other proceedings); if you have consented to the disclosure, including for your medical treatment; or if it is used for other scientific research, as allowed by federal regulations protecting research subjects.

If you have any questions, concerns, or complaints about this interview, you may contact the Principal Investigator, Dr. Laura Y. Cabrera at 517-355-7553 or at laura.cabrera@hc.msu.edu. If you have any concerns or complaints about your rights as a research participant and/or your experience while participating in this study, contact the Michigan State University’s Human Research Protection Program by phone (517-355-2180), fax (517-432-4503), e-mail (irb@msu.edu) or regular mail (4000 Collins Rd, Suite 136; Lansing, MI 48910).

You indicate that you voluntarily agree to participate in this research study by proceeding with the interview.

**Consent Letter for Public Interviews**

You are being asked to participate in a research study. The purpose of the study is to identify concerns, beliefs, and attitudes about different types of treatments for clinical depression. This initial stage of our study will help us better understand what members of the public without any mental health disorders think about therapies that treat depression by using electrical stimulation to affect and modify brain function. In addition to interviews with members of the public, we are also interviewing psychiatrists as well as patients with depression.

In our interviews of people with depression, we are deeply interested in your views about therapies that use electrical stimulation to treat depression, including how you perceive their benefits, risks, ethical considerations around their use and how you think these therapies may be developed further.

We want to achieve as much depth of understanding as possible in our interviews. Your honest and thoughtful answers today will also help us design a standardized survey about depression treatments that use electrical stimulation, which we will be administering to a national sample of people.

For the interview, you will be asked to answer some questions. It should take about 60 minutes. Your participation is voluntary. If you not do want to answer any of the questions, please let me know. You can also ask me to stop at any time. You must be 18 or older to participate. Is it okay if I record our interview?

This research is covered by a Certificate of Confidentiality from the National Institutes of Health. The researchers with this Certificate may not disclose or use information, documents, or biospecimens that may identify you in any federal, state, or local civil, criminal, administrative, legislative, or other action, suit, or proceeding, or be used as evidence, for example, if there is a court subpoena, unless you have consented for this use. Information, documents, or biospecimens protected by this Certificate cannot be disclosed to anyone else who is not connected with the research except, if there is a federal, state, or local law that requires disclosure (such as to report child abuse or communicable diseases but not for federal, state, or local civil, criminal, administrative, legislative, or other proceedings); if you have consented to the disclosure, including for your medical treatment; or if it is used for other scientific research, as allowed by federal regulations protecting research subjects.

If you have any questions, concerns, or complaints about this interview, you may contact the Principal Investigator, Dr. Laura Y. Cabrera at 517-355-7553 or at laura.cabrera@hc.msu.edu. If you have any concerns or complaints about your rights as a research participant and/or your experience while participating in this study, contact the Michigan State University’s Human Research Protection Program by phone (517-355-2180), fax (517-432-4503), e-mail (irb@msu.edu) or regular mail (4000 Collins Rd, Suite 136; Lansing, MI 48910).

You indicate that you voluntarily agree to participate in this research study by proceeding with the interview.

**Semi-Structured Interview Guide for Patients**

[*record the following on each respondent*]

age: ____ race: _________________ ethnicity: _____________ gender: ____

marital status: _______________ education: ________________ income: ____________

Thank you for agreeing to participate in this interview. As we explained in the consent letter, this initial stage of our study will help us better understand what people with depression think about therapies that treat depression by using electrical stimulation to affect and modify brain function. In addition to interviews with people with depression, we are also interviewing psychiatrists as well as members of the public without depression.

In our interviews of people with depression, we are deeply interested in your views about therapies that use electrical stimulation to treat depression, including how you perceive their benefits, risks, ethical considerations around their use and how you think these therapies may be developed further.

We want to achieve as much depth of understanding as possible in our interviews. Your honest and thoughtful answers today will also help us design a standardized survey about depression treatments that use electrical stimulation, which we will be administering to a national sample of people.

Before, we begin I’d just like you to know that this will be a semi-structured interview. That is, I have a few groups of questions I’d like you to address. And we will generally follow the order of the questions on my guide. But, I will let you guide the exact order and pacing, since I am most interested in your own perceptions about these interventions.

Okay, let’s get started with a few questions about your background and experiences.

Your Background and Experiences

When were you first clinically diagnosed with depression?

What was that experience like?

What sort of concerns did you have?

Have you been diagnosed with any other mental health conditions?

What depression treatments have you tried so far?

[*half of our patients will have PEI experience, and half will not have PEI experience*]

How effective do you think it/they have been?

[*if subject has tried a PEI, jump to prompt A; then return to ask about knowledge regarding other PEIs*]

Now, let’s shift to the main topic of our interview today.

Your Familiarity and Experience with PEIs

Before reading the information we sent you, what did you know about treatments that use electrical stimulation to treat depression? Which specific treatments were you familiar with (for example, ECT, TMS, DBS, or ABI)?

Have you ever read anything, watched anything, or talked to anyone that has influenced how you view these procedures?

Okay, now let’s take a look at the information we sent you about the different treatments we’re focusing on for this interview. Do you have any questions?

How would you explain [*PEI*] to somebody else: [*insert each of the PEIs and get description*]

electroconvulsive therapy or ECT?

transcranial magnetic stimulation or TMS?

deep brain stimulation or DBS?

adaptive brain implants or ABI?

ECT? _______ TMS? _______ DBS? _______ ABI? _______

[*note the ones that patient has experience with and be sure to include them in subsequent questions as indicated. For those with no experience, ask about familiarity, and make sure we cover all 4 PEIs*]

A. **[*if a patient has undergone therapy with a PEI*]**

Tell me about your experience being treated with [*ECT/TMS/DBS/ABI*].

How did you first learn about the therapy?

How did you decide to try [*ECT/TMS/DBS/ABI*]?

How does treatment with [*ECT/TMS/DBS/ABI*] compare to other therapies you have tried?

B. **[*if a patient has not undergone therapy with a PEI*]**

What do you know about the kinds of therapy that are available for depression?

When you are thinking about using a therapy, what kinds of questions or concerns do you have?

How do you find information about the therapy?

Would you ever consider using an intervention that stimulates the brain with electrical signals as its way to treat your depression, such as ECT, DBS, or TMS?

Under what circumstances would you consider it?

Okay, now let’s discuss in greater detail what you think about some of these therapies. We’ll go through a few questions about [*insert PEI*] and then go back and ask the same questions about [*insert second PEI*].

Your Views about the Group of PEIs in This Study

Think about [*the PEI they are the most familiar with: ECT/TMS/DBS/ABI*]. [*If we get too many interviews with only one or two, we can try to ask about others if they have at least some knowledge about them to get some balance.*]

Next we have some questions about the effects of [*ECT/TMS/DBS/ABI*]:

What do you think are the benefits of [*ECT/TMS/DBS/ABI*] compared to psychotherapy or taking pharmaceuticals? [*note similarities and differences*]

What do you think are the risks of [*ECT/TMS/DBS/ABI*] compared to psychotherapy or taking pharmaceuticals? [*note similarities and differences*]

What do you think are the side effects [*ECT/TMS/DBS/ABI*] [*note similarities and differences, how serious, and how likely*]

How do you think the potential side effects are similar to or different from the potential side effects of undergoing psychotherapy? Why? How so?

How do you think the potential side effects are similar to or different from the potential side effects of taking pharmaceuticals? Why? How so?

How do you think the potential side effects are similar to or different from the potential side effects of DBS for essential tremor? Why? How so?

How do you think the potential side effects are similar to or different from the potential side effects of inserting a stent in the brain to prevent blocked arteries? Why? How so?

Do you think [*ECT/TMS/DBS/ABI*] has the potential to affect a patient’s personality or sense of self? Why? How so?

How do you think this potential compares to that of the same potential undergoing psychotherapy has? Why? How so?

How do you think this potential compares to that of the same potential taking pharmaceuticals has? Why? How so?

How do you think this potential compares to that of DBS for essential tremor? Why? How so?

How do you think this potential compares to that of inserting a stent in the brain to prevent blocked arteries? Why? How so?

How would you describe how invasive [*ECT/TMS/DBS/ABI*] is? Why? In what sense?

Do you think [*ECT/TMS/DBS/ABI*] is more or less invasive than undergoing psychotherapy?

Do you think [*ECT/TMS/DBS/ABI*] is more or less invasive than taking pharmaceuticals? Why? How so?

Do you think it is more or less invasive than use of DBS for essential tremor? Why? How so?

Do you think it is more or less invasive than the insertion of a stent in the brain to prevent blocked arteries? Why? How so?

Right now, DBS usually requires patients to go to their doctor if the level of stimulation needs to be increased or decreased. Do you think that, instead, patients should be able adjust their own level of stimulation? Why/why not?

There is also interest in developing an adaptive version of DBS, in which the implant can detect brain activity and adjust the level of stimulation automatically, if necessary. Do you think an adaptive brain implant has different risks and benefits than current DBS? Why? How so?

[*if they are familiar with another PEI ask this in the second round of questions, and if they compare it to the first PEI do not ask about the other comparisons. If not familiar with any in particular ask these question*]:

Of [*ECT/TMS/DBS/ABI*], which do you consider the most invasive treatment? Why?

Of [*ECT/TMS/DBS/ABI*], which do you consider the least invasive treatment? Why?

Of [*ECT/TMS/DBS/ABI*], which do you consider to have the best risk/benefit ratio? Why?

Do you have any concerns about practical barriers to accessing these therapies, if patients want to use them? Are there things that you think might keep people from being able to use these treatments? (e.g., distance, waiting times, side effects, transportation)?

What do you see as the most important ethical questions we should answer about using [*ECT/TMS/DBS/ABI*] for treating psychiatric disorders? Why? How so?

To what extent do these questions vary depending up the PEI in focus? Why? How so?

To what do you have concerns or expectations regarding PEIs that are different from those about psychotherapy or pharmaceutical treatments? Why? How so?

To what extent are these questions similar to or different from ones you would have about the insertion of a stent in the brain to prevent blocked arteries? Why? How so?

Wrap-Up and Thank You

Thank you for participating in our study. Your participation in this interview is very important to us. Your answers will help us advance knowledge about how patients view therapies that use electrical stimulation to treat depression. Your answers also will help us develop the national surveys in the next stage of the project.

[*If in person*] Thank you again for your time. Here is your gift card for participating in the study.

[*If by Zoom*] Thank you again for your time. Can you please provide me with the mailing address or email where we can send you the gift card for participating in the study?

**Common Probe Questions**

Clarification

What do you mean?

Tell me what you mean when you say [. . .].

It sounds like you are saying, “. . . .” Is that a fair summary?

Details and Depth

Tell me more about that.

Can you give me an example?

Can you tell me more about that?

Thought Process

Why do you think that?

Why is that important?

What do you mean?

What do you think that stands out in your memory?

Why do you think you noticed that?

Why does that matter?

How did you feel about that?

**Semi-Structured Interview Guide for Public**

[*record the following on each respondent*]

age: ____ race: _________________ ethnicity: _____________ gender: ____

marital status: _______________ education: ________________ income: ____________

Thank you for agreeing to participate in this interview. As we explained in the consent letter, this initial stage of our study will help us better understand what members of the public without any mental health disorders think about therapies that treat depression by using electrical stimulation to affect and modify brain function. In addition to interviews with members of the public, we are also interviewing psychiatrists as well as patients with depression.

In our interviews of members of the public, we are deeply interested in your views about therapies that use electrical stimulation to treat depression, including how you perceive their benefits, risks, ethical considerations around their use and how you think these therapies may be developed further.

We want to achieve as much depth of understanding as possible in our interviews. Your honest and thoughtful answers today also will help us design a standardized survey about depression treatments that use electrical stimulation, which we will be administering to a national sample of people.

Before, we begin I’d just like you to know that this will be a semi-structured interview. That is, I have a few groups of questions I’d like you to address. And we will generally follow the order of the questions on my guide. But, I will let you guide the exact order and pacing, since I am most interested in your own perceptions about these interventions.

Okay, let’s get started with a few questions about your background and experiences.

Your Background and Experiences

Have you ever been diagnosed with a mental health condition?

What do you think causes depression?

[*only if participant says they don’t know and asks what depression is*] Depression is a mental illness that involves a depressed mood or loss of enjoyment in a person’s life and activities. It may also involve changes in appetite or sleep patterns, and other emotional and cognitive difficulties. Doctors don’t know what causes depression, though most people agree that it involves changes to brain activity and is usually associated with personal or social stresses.

How effective do you think current treatments for depression are?

Have you ever assisted someone with a mental health disorder as a caregiver?

[*half of the general public interviewees will have experience caring for someone with a mental health disorder, and half will not have such experience*]

[*if yes*] Can you tell us more about what that was like? What sorts of help did you give them?

Now, let’s shift to the main topic of our interview today.

Your Familiarity and Experience with PEIs

Before reading the information we sent you, what did you know about treatments that use electrical stimulation to treat depression? Which specific treatments were you familiar with (for example, ECT, TMS, DBS, or ABI)?

Have you ever read anything, watched anything, or talked to anyone that has influenced how you view these procedures?

Okay, now let’s take a look at the information we sent you about the different treatments we’re focusing on for this interview. Do you have any questions?

How would you explain [*PEI*] to somebody else: [*insert each of the PEIs and get description*]

electroconvulsive therapy or ECT?

transcranial magnetic stimulation or TMS?

deep brain stimulation or DBS?

adaptive brain implants or ABI?

ECT? _______ TMS? _______ DBS? _______ ABI? _______

[*note the ones that person has experience with and be sure to include them in subsequent questions as indicated. For those with no experience, ask about familiarity, and make sure we cover all 4 PEIs*]

A. **[*if a member of the public is familiar with a PEI*]**

Tell me about how you learned about [*ECT/TMS/DBS/ABI*].

B. **[*if a member of the public is not familiar with a PEI*]**

Where have you read information about therapies for depression?

What do you think about using an intervention that stimulates the brain with electrical signals as a way to treat depression, such as ECT, DBS, or TMS?

Okay, now let’s discuss in greater detail what you think about some of these therapies. We’ll go through a few questions about [*insert PEI*] and then go back and ask the same questions about [*insert second PEI*].

Your Views about the Group of PEIs in This Study

Think about [*the PEI they are the most familiar with: ECT/TMS/DBS/ABI*]. [*If we get too many interviews with only one or two, we can try to ask about others if they have at least some knowledge about them to get some balance.*]

Next we have some questions about the effects of [*ECT/TMS/DBS/ABI*]:

What do you think are the benefits of [*ECT/TMS/DBS/ABI*] compared to psychotherapy or taking pharmaceuticals? [*note similarities and differences*]

What do you think are the risks of [*ECT/TMS/DBS/ABI*] compared to psychotherapy or taking pharmaceuticals? [*note similarities and differences*]

What do you think are the side effects [*ECT/TMS/DBS/ABI*] [*note similarities and differences, how serious, and how likely*]

How do you think the potential side effects are similar to or different from the potential side effects of undergoing psychotherapy? Why? How so?

How do you think the potential side effects are similar to or different from the potential side effects of taking pharmaceuticals? Why? How so?

How do you think the potential side effects are similar to or different from the potential side effects of DBS for essential tremor? Why? How so?

How do you think the potential side effects are similar to or different from the potential side effects of inserting a stent in the brain to prevent blocked arteries? Why? How so?

Do you think [*ECT/TMS/DBS/ABI*] has the potential to affect a patient’s personality or sense of self? Why? How so?

How do you think this potential compares to that of the same potential undergoing psychotherapy has? Why? How so?

How do you think this potential compares to that of the same potential taking pharmaceuticals has? Why? How so?

How do you think this potential compares to that of DBS for essential tremor? Why? How so?

How do you think this potential compares to that of inserting a stent in the brain to prevent blocked arteries? Why? How so?

How would you describe how invasive [*ECT/TMS/DBS/ABI*] is? Why? In what sense?

Do you think [*ECT/TMS/DBS/ABI*] is more or less invasive than undergoing psychotherapy?

Do you think [*ECT/TMS/DBS/ABI*] is more or less invasive than taking pharmaceuticals? Why? How so?

Do you think it is more or less invasive than use of DBS for essential tremor? Why? How so?

Do you think it is more or less invasive than the insertion of a stent in the brain to prevent blocked arteries? Why? How so?

Right now, DBS usually requires patients to go to their doctor if the level of stimulation needs to be increased or decreased. Do you think that, instead, patients should be able adjust their own level of stimulation? Why/why not?

There is also interest in developing an adaptive version of DBS, in which the implant can detect brain activity and adjust the level of stimulation automatically, if necessary. Do you think an adaptive brain implant has different risks and benefits than current DBS? Why? How so?

[*if they are familiar with another PEI ask this in the second round of questions, and if they compare it to the first PEI do not ask about the other comparisons. If not familiar with any in particular ask these question*]:

Of [*ECT/TMS/DBS/ABI*], which do you consider the most invasive treatment? Why?

Of [*ECT/TMS/DBS/ABI*], which do you consider the least invasive treatment? Why?

Of [*ECT/TMS/DBS/ABI*], which do you consider to have the best risk/benefit ratio? Why?

Do you have any concerns about practical barriers to accessing these therapies, if patients want to use them? Are there things that you think might keep people from being able to use these treatments? (e.g., distance, waiting times, side effects, transportation)?

What do you see as the most important ethical questions we should answer about using [*ECT/TMS/DBS/ABI*] for treating psychiatric disorders? Why? How so?

To what extent do these questions vary depending up the PEI in focus? Why? How so?

To what do you have concerns or expectations regarding PEIs that are different from those about psychotherapy or pharmaceutical treatments? Why? How so?

To what extent are these questions similar to or different from ones you would have about the insertion of a stent in the brain to prevent blocked arteries? Why? How so?

If you or a loved one had disabling or life-threatening depression, would you consider use of [*ECT/TMS/DBS/ABI*] if it would bring relief/save a life? Why? Why not?

Wrap-Up and Thank You

Thank you for participating in our study. Your participation in this interview is very important to us. Your answers will help us advance knowledge about how members of the public view therapies that use electrical stimulation to treat depression. Your answers also will help us develop the national surveys in the next stage of the project.

[*If in person*] Thank you again for your time. Here is your gift card for participating in the study.

[*If by Zoom*] Thank you again for your time. Can you please provide me with the mailing address or email where we can send you the gift card for participating in the study?

**Common Probe Questions**

Clarification

What do you mean?

Tell me what you mean when you say [. . .].

It sounds like you are saying, “. . . .” Is that a fair summary?

Details and Depth

Tell me more about that.

Can you give me an example?

Can you tell me more about that?

Thought Process

Why do you think that?

Why is that important?

What do you mean?

What do you think that stands out in your memory?

Why do you think you noticed that?

Why does that matter?

How did you feel about that?

**Table SDC3**. Predominant attitude toward PEIs by participant

**Participant # ECT TMS DBS**

Patient1 negative positive negative

Patient2 negative positive --

Patient3 --* -- positive

Patient4 negative cautionary cautionary

Patient5 cautionary cautionary --

Patient6 cautionary cautionary cautionary

Patient7 negative cautionary negative

Patient8 cautionary negative cautionary

Patient9 positive -- negative

Patient10 cautionary -- negative

Patient11 cautionary -- --

Patient12 cautionary -- cautionary

Patient13 cautionary cautionary negative

Patient14 negative -- positive

Patient15 -- cautionary negative

Patient16 cautionary -- positive

**Participant # ECT TMS DBS**

Public1 cautionary -- positive

Public2 cautionary -- cautionary

Public3 positive cautionary cautionary

Public4 cautionary cautionary cautionary

Public5 -- cautionary --

Public6 negative -- --

Public7 positive -- cautionary

Public8 positive -- --

Public9 -- -- cautionary

Public10 negative cautionary cautionary

Public11 -- positive cautionary

Public12 -- positive positive

Public13 negative positive positive

Public14 -- -- cautionary

Public15 -- -- positive

Public16 -- -- negative

* Participant was not asked about this PEI and/or did not voice any discernable attitude about it.
